# Chrononutrition in Chronic Kidney Disease

**DOI:** 10.3390/nu17030389

**Published:** 2025-01-22

**Authors:** Pilar C. Castro-Mata, Alfonso M. Cueto-Manzano, Barbara Vizmanos, Ailema González-Ortiz, Alejandra Betancourt-Núñez, Fabiola Martín-del-Campo

**Affiliations:** 1 Medical Research Unit of Renal Diseases, Specialties Hospital, Western National Medical Center, Mexican Institute of Social Security (IMSS), Guadalajara 44320, Mexico; pilar.castro3712@alumnos.udg.mx (P.C.C.-M.); a_cueto_manzano@hotmail.com (A.M.C.-M.); 2 PhD Program in Translational Nutrition Sciences, Department of Human Reproduction, Child Growth and Development, University Center of Health Sciences (CUCS), Guadalajara 44340, Mexico; bvizmanos@yahoo.com.mx (B.V.); alejandra.bnunez@academicos.udg.mx (A.B.-N.); 3 Translational Research Center, National Institute of Pediatrics, Mexico City 04530, Mexico; ailejgo@gmail.com

**Keywords:** chrononutrition, chronic kidney disease, meal timing, nutrition

## Abstract

Chrononutrition, the study of the interaction between biological rhythms and nutrition, has emerged as a promising field for addressing metabolic health. However, its role in chronic kidney disease (CKD) remains underexplored. CKD patients often experience circadian disruptions due to renal, metabolic, treatment-related, and lifestyle factors, which may influence their nutritional status and clinical outcomes. Objective: to synthesize and analyze the existing evidence on chrononutrition in CKD patients, identify knowledge gaps, and propose directions for future research across different stages of CKD. Initially, this review contextualizes circadian physiology, alignment, and chronodisruption to explore such factors in CKD patients, focusing on chrononutrition variables already studied in the general population. We discuss how dietary timing and habit adjustments could influence CKD clinical outcomes, offering insights into circadian impacts on disease management. This new approach could optimize patient care, encouraging further research, particularly in the development of personalized strategies for different stages of the disease.

## 1. Background

Chronobiology is a field of biology dedicated to the study of temporal rhythms in living organisms and their synchronization with environmental cycles. The 24 h circadian rhythm demonstrates how internal biological processes are aligned with external factors, such as the cycles of light and darkness [1]. A growing body of research in chronobiology offers promising insights into improving human health by enhancing the understanding of how the synchronization of biological clocks with environmental and lifestyle factors can influence overall well-being [2].

Nutrition is pivotal in human chronobiology, characterized by a bidirectional relationship [3]. On the one hand, feeding behaviors can serve as powerful zeitgebers, or time cues, that help regulate circadian rhythms [4]. On the other hand, disruptions in chronobiology, such as those caused by irregular sleep patterns or misalignment with the natural light-dark cycle, can significantly impact individual’s nutritional status and their metabolic health [5].

Despite these important connections, the interplay between chronobiology and nutrition has not been thoroughly explored in patients with chronic kidney disease (CKD), likely due to the complex and multifactorial nature of the disease. By contrast, this relationship has been more extensively studied in other conditions, such as obesity, diabetes, and cardiovascular diseases, where disruptions in circadian rhythms and misaligned meal timing have been linked to metabolic dysregulation, weight gain, and increased cardiovascular risk [6,7]. Nevertheless, patients who live with CKD often experience a range of metabolic disturbances [8], sleep disorders, and altered circadian rhythms [9,10] which may, in turn, influence their nutritional status and clinical outcomes [11].

This review contextualizes elements of circadian physiology and alignment and chronodisruption to allow for a deeper exploration, specifically in CKD patients. By examining chronodisruption and its impacts, how disturbances in circadian rhythms affect this population could be better understood. Additionally, key chrononutrition variables previously studied in the general population are assessed in terms of their applications on CKD patients. Finally, the review will discuss how adjustments in dietary habits could potentially influence clinical outcomes in CKD, providing insights into the role of timing and nutrition in disease management.

The objective of this review is to synthesize and analyze the existing evidence on chrononutrition in CKD patients, identifying knowledge gaps and proposing directions for future research to better understand how dietary timing interventions may improve nutritional status and clinical outcomes across different stages of CKD.

### 1.1. Circadian Physiology

The circadian rhythm is a daily oscillation of molecular, physiological, and behavioral processes that allows organisms to anticipate and prepare for changes in bodily functions over the 24 h day–night cycle [12]. The circadian system is organized into a hierarchical network, featuring a central clock located in the suprachiasmatic nucleus (SCN) of the hypothalamus, and peripheral clocks situated in various organs and tissues, including the liver, heart, kidneys, intestines, and adipose tissue [12,13].

The molecular clock is a transcriptional/translational feedback loop composed of two heterodimers, one positive (BMAL1, CLOCK) and one negative (PER, CRY), which act either to increase or decrease the transcription of circadian clock genes by acting on the E-box response element [14]. These mechanisms regulate specific gene targets in tissues that control rhythmic physiological and behavioral outputs [15]. Through these rhythmic outputs, the circadian system governs processes related to energy balance, feeding–fasting rhythms, the sleep–wake cycle, and the metabolic response to food intake, blood pressure, body temperature, and metabolism [16]. This adaptation seems to provide the advantage of anticipating daily changes in the environment [13,15].

Molecular and physiological rhythms play a key role in regulating essential bodily functions and influence individual variations in sleep–wake patterns and daily activity preferences, known as chronotypes (CT). CT, defined as the individual awake/sleep behavior, are distributed along a continuum, ranging from extremely morning-oriented to extremely evening-oriented individuals. Broadly, CT is categorized into three types: morning, evening, and intermediate. Individuals without a strong circadian preference are classified as intermediate types because they exhibit characteristics of both morning and evening CT [17].

### 1.2. Circadian Alignment and Chronodisruption

The circadian system works through synchronization signals, or zeitgebers, which refer to any external input or cue that can synchronize an endogenous rhythm. Consequently, a continuous relationship is established and maintained between the external factors and the organism’s endogenous rhythm, ensuring the correspondence between geological and biological times [18].

The primary zeitgeber for the central circadian clock is light, which triggers the light/dark cycle and synchronizes peripheral clocks to a 24-h rhythm. While the SCN is driven by photic signals, other non-photic external signals such as exercise and food intake are powerful zeitgebers for peripheral clocks, but not for the central circadian clock [18,19]. Accordingly, the non-photic stimuli reset the phase of peripheral clocks without changing the phase of the central clock, leading to the potential for misalignment between central and peripheral rhythms [2].

**Figure 1:**
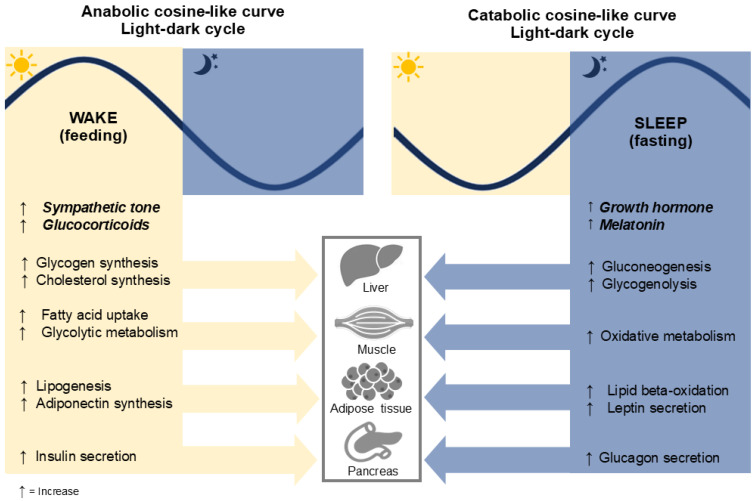
Metabolic processes in alignment with the light-dark cycle. The figure illustrates some metabolic processes that occur during wakefulness and sleep, regulated by the circadian rhythm and light-dark cycle. During the wake phase (feeding), anabolic processes dominate, including increased glycogen and cholesterol synthesis, fatty acid uptake, glycolytic metabolism, lipogenesis, adiponectin synthesis, and insulin secretion. In contrast, during the sleep phase (fasting), catabolic processes prevail, characterized by elevated growth hormone and melatonin levels, increased gluconeogenesis, glycogenolysis, oxidative metabolism, lipid beta-oxidation, leptin secretion, and glucagon secretion. These processes are coordinated across key metabolic organs, including the liver, muscles, adipose tissue, and pancreas.

When the central and peripheral circadian clocks are properly aligned with the environment, the circadian system regulates metabolic responses in peripheral tissues according to the light/dark cycle. The circadian rhythm follows a cosine-like curve, where the peaks and troughs correspond to different metabolic processes (Figure 1). These are divided into two main phases: anabolism (during the day, associated with feeding) and catabolism (during the night, associated with fasting). For example, during the day, the liver clock promotes glycogen and cholesterol synthesis to store energy. At night, the same clock activates gluconeogenesis and glycogenolysis, processes that mobilize stored energy to maintain metabolic balance during the fasting period [5,20,21].

Chronodisruption can occur at various levels, including misalignment between external and internal time, desynchrony among tissue clocks, and phase incoherence within tissues [22].

The proper functioning of peripheral clocks keeps metabolic processes in synchrony with the environment, which is critical for maintaining a healthy organism. Circadian alterations are characterized by a disrupted rhythm that can be short-term or long-term. A chronodisturbance is a temporary disruption of the circadian rhythm that leads to an adaptive response, helping to minimize its negative effects. While chronodisruption refers to a long-term alteration that may cause disease, driven or mediated by one or more chronodisruptors that constantly desynchronize the internal and external rhythms [19].

The synchrony of circadian rhythms among tissues and organs appears to be critical for health. Several studies have evidenced the relationship between the misalignment of sleeping and eating schedules and its association with obesity, diabetes, and metabolic syndrome [3,18,23]. This has been widely explored in shift workers, who continuously move their rest/work schedules on irregular periods even on different day/night turns; this population has been associated with various metabolic disorders [24] and chronic diseases [25].

Furthermore, some evidence suggests that individuals with an evening CT are more susceptible to chronodisruption, as their preferred activity times often conflict with societal schedules, leading to misalignment with their internal clocks. This misalignment has been associated with suboptimal lifestyle habits and adverse health outcomes compared to morning CT [26,27,28].

The timing of meals is a modifiable temporal zeitgeber for the circadian system, particularly the peripheral clocks of tissues or organs related to metabolism (gut, liver, adipose tissue) [29] but not for the central clock [30,31], thus highlighting the importance of circadian rhythms in metabolic health.

### 1.3. Chronodisruptors in Chronic Kidney Disease

In CKD, the circadian rhythm can be disrupted by multiple factors, which have been studied mainly in murine models under controlled environments [13]. Considering the complexity and interrelated nature of chronobiological alterations in CKD, research in humans has begun to explore these disruptions, given the challenge of studying them under multifactorial uncontrolled conditions. Despite these challenges, it is essential to consider these disruptors as potential therapeutic targets [13,19].

Some chronodisruptors in CKD have been previously described and can be grouped into four categories, as follows: kidney-specific alterations, renal replacement therapy-related, psychological-and social-related alterations, and environmental and lifestyle.

#### 1.3.1. Renal-Specific Alterations

Some renal functions, such as diuresis, glomerular filtration rate (GFR), renal plasma flow, and others, follow circadian rhythms that have been previously described [32,33,34]. Reduced renal function is associated with alterations that disrupt physiological timing, acting as chronodisruptors. These chronodisruptors may include uremic toxins and altered circadian rhythms that may be associated with CKD progression [19]. These changes interfere with the body’s natural rhythms, exacerbating circadian misalignment.

Nocturia, for example, represents a reversal of circadian regulation of urine output, characterized by nighttime waking more than twice to urinate and excreting half of the total urinary sodium overnight, which physiologically should be excreted during the day. This frequent condition in CKD patients is associated with poor sleep and increased daytime arterial blood pressure [35], disrupting the normal circadian regulation of fluid balance.

Regarding alterations in kidney structure, albuminuria itself may potentially act as a chronodisruptor, even when kidney function is preserved. In patients with nephrotic syndrome and overall preserved GFR (75 mL/min/1.73 m^2^), blood pressure ratios during sleep/wake cycles were higher than in healthy controls, a phenomenon that was reversed upon remission of proteinuria [36]. This elevation in nighttime blood pressure further destabilizes circadian homeostasis. Renal fibrosis has been reported, because of central clock alterations in hamsters, leading to renal damage and premature death [37]. TGFβ-dependent renal fibrosis was observed in CLOCK-deficient mice compared to wild-type counterparts, with increased fibrosis attributed to elevated COX2 expression [38]. Such fibrosis reflects the impact of circadian dysregulation on renal structural integrity, reinforcing chronodisruption effects.

Changes in blood pressure timing, such as reverse dipping (nighttime blood pressure peak) for systolic pressure and episodes of daytime hypotension [34,39], illustrate circadian misalignment in vascular control. These alterations contribute to chronodisruption by disturbing the expected daily rhythm of blood pressure regulation.

Additionally, erythropoietin deficiency seems to have a role in the dysregulation of melatonin metabolism in CKD [40] and has been associated with sleep alterations. Although the relationship between erythropoietin and melatonin is still under study, regular erythropoietin administration leads to the correction of anemia and partial restoration of the nocturnal rise in melatonin in murine models. This observation points to the possible role of EPO deficiency in the pathogenesis of melatonin dysregulation in CKD [40,41].

#### 1.3.2. Renal Replacement Therapy-Related Alterations

Hemodialysis (HD) and peritoneal dialysis (PD) themselves can lead to chronic disturbances due to various factors [42].

In HD, chronic temperature rises may also disrupt normal sleep rhythms. The heat load from the dialysate can increase body temperature, potentially enhancing daytime sleep propensity by activating cooling mechanisms associated with sleep onset [9]. Dysregulation of melatonin secretion could partially explain the symptoms in these patients or serve as a biomarker of disruption. In an uncontrolled study, nocturnal HD improved melatonin levels as well as sleep efficiency and quality, potentially enhancing the quality of life for patients undergoing HD [9].

Finally, the day and time of dialysis, either on PD or HD, often overrides natural light-dark cycles. This irregular exposure to light, a key regulator of circadian rhythms, combined with the demands of dialysis, creates a significant misalignment between internal biological clocks and environmental cues [43]. Specifically, CKD patients undergoing HD with morning CT were associated with chronodisruption and alterations in their quality of life [44].

In patients undergoing PD, the presence of peritoneal fluid may elevate the risk of sleep apnea, additionally, the PD treatment schedule typically includes exchanges around midnight and early morning, this routine can further limit their total sleep duration and affects the circadian natural rhythm [45].

#### 1.3.3. Psychological- and Social-Related Alterations

Living with CKD, and especially the dialysis procedure itself, are significant physical and psychological stressors that negatively affect every aspect of a patient’s life [46]. Socio-economic factors like unemployment and reduced work capacity are prevalent in CKD patients, especially those in advanced stages. Furthermore, rigorous treatment routines (multiple medical visits, polypharmacy, renal replacement therapies, etc.) often lead to significant stress and emotional strain, triggering anxiety, depression, and fatigue [47]. These factors consequently bring excessive daytime sleepiness mixed with sleep disturbances, reported by 30% to 80% of individuals with end-stage kidney disease [9].

#### 1.3.4. Environmental and Lifestyle Determinants

Environmental and cultural factors such as tradition, religion, history, and socioeconomic status influence daily routines, including sleep–wake cycles and lifestyle habits. In patients with CKD, these factors interact with the challenges of disease management. A key difference in this population is the limited relevance of the typical workday–weekend structure. Due to high unemployment rates, often a result of disease-related disability, many CKD patients are not bound by regular work schedules. Instead, their routines are shaped by dialysis treatments, which sometimes follow irregular shift rotations [44].

These environmental and cultural challenges accentuate the distinctive chronodisruptors faced by CKD patients, which require further investigation to improve the management of their circadian health [19].

Physical activity also could play a role as a chronodisruptor in CKD patients. Due to fatigue, muscle wasting, and reduced physical capacity, many patients experience lower levels of physical activity, which further disturbs circadian regulation. Physical exercise is a known synchronizer of circadian rhythms, and its absence or irregularity can worsen chronodisruption [48]. The lack of consistent physical activity, especially on dialysis days, adds another layer of complexity to maintaining circadian alignment in CKD patients.

Diet itself can act as a chronodisruptor in the general population, due to the complex interaction between food intake and the body’s internal circadian rhythms [49]. In CKD, disruptions in meal timing, frequency, and composition (often influenced by dietary restrictions related to the disease) can disrupt the body’s natural biological clocks. This issue will be explored in more detail below.

### 1.4. Chrononutrition

There is increasing evidence that highlights the importance of not only the quantity and type of food eaten, but also the timing of meals. The timing of food intake plays a significant role in maintaining energy balance, regulating metabolism, and promoting optimal health [31,50]. Conversely, consuming nutrients at inappropriate times of the day can lead to circadian misalignment, associated with metabolic imbalances [16]. Furthermore, the metabolic demands of CKD patients, combined with restrictions on protein, sodium, and fluid intake, can exacerbate this misalignment, resulting in chronodisruption.

This emerging research suggests that aligning the time of the meals with circadian rhythms could improve metabolic outcomes in general populations and reduce metabolic diseases, but this topic has been scarcely described in CKD patients.

Chrononutrition is the study of the interaction between biological rhythms and nutrition, as well as their relationship with health [18]. Chrononutrition analyzes three different dimensions of eating behavior, including the timing, frequency, and regularity of meals [18,49,51].

**Figure 2:**
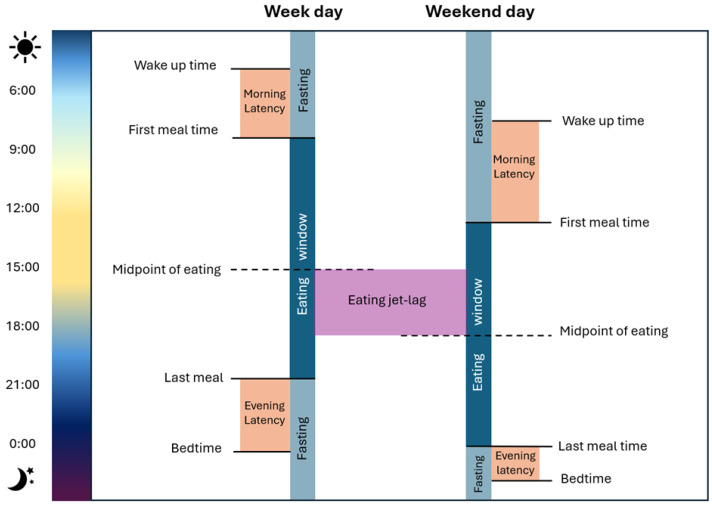
Chrononutrition variables. The figure illustrates the main variables studied in chrononutrition, including wake-up time, first and last meal timing, eating window, fasting periods, morning and evening latencies, and eating jet lag.

For a more precise association, multiple chrononutrition variables/terminology have been identified (Figure 2). Among the most recently studied are the timing of meals in relation to the day/night cycle [52], morning and evening latencies (from wake-up time until the first eating event, and from the last eating event until bedtime) [28], eating windows, the midpoint of eating, and meal timing misalignment between weekdays and weekends, known as eating jet lag [53]. These variables have been associated with both favorable and unfavorable metabolic outcomes, such as glucose regulation, lipid profile, and overall nutritional status. In the following sections, these variables will be explored in detail, drawing on specific findings in CKD patients.

These chrononutrition variables have the potential to develop time related interventions aimed at improving the overall health status of patients living with CKD. To support this, we conducted research to identify the existing evidence on chrononutrition interventions within this population, providing a foundation for further exploration and application in clinical practice.

## 2. Methods

The research question guiding this narrative review is: “In people with CKD, does chrononutrition influence nutritional status?”. The PICO framework was defined as follows: P (Population): Adult patients with CKD at different stages (early stage, end-stage, HD, DP, or transplant). I (Intervention/Exposition): Chrononutrition (eating/fasting windows, timing of food/supplement intake, CT-based meal timing). C (Comparator): Not applicable (no comparison included). O (Outcome): Nutritional status.

The search strategy was conducted using a combination of MeSH terms and keywords aligned with the research question. Terms related to the population included “chronic kidney disease”, “CKD”, “renal insufficiency”, “hemodialysis”, “peritoneal dialysis”, and “kidney transplantation”. For the intervention/exposition, terms such as “chrononutrition”, “meal timing”, “eating window”, “time-restricted feeding”, “chronotype”, and “eating jet-lag” were used. Outcomes were identified with terms like “nutritional status”, “protein-energy wasting (PEW)”, “PEW”, and “body composition”. Boolean operators (AND, OR, NOR) were applied to combine terms. The search was conducted across PubMed and Google Scholar, including articles published up to December 2024.

Inclusion and exclusion criteria were established to ensure the relevance and quality of the selected studies. Inclusion criteria comprised studies involving adult CKD patients, clinical trials, pilot studies, cohort studies, and cross-sectional studies focusing on chrononutrition strategies. Studies needed to report outcomes related to nutritional status, metabolic parameters and be published in peer-reviewed journals in English or Spanish. The exclusion criteria included studies not involving CKD patients, reviews, editorials, or opinion pieces without primary data, studies focusing on general dietary interventions without a chrononutrition component, and animal studies. Details of the complete search strategy can be found in the Supplementary Table S1.

## 3. Results

A total of 136 studies were identified through the initial search; 60 studies were included after the screening by title and abstract, however, only 11 met the inclusion criteria and were directly relevant to chrononutrition exposure on CKD patients. Regarding the type of evidence, we found mainly observational studies on populations practicing Ramadan fasting. This evidence shows specifically the effects of fasting on CKD patients of all stages. Also, we found pilot studies that involved chrononutrition interventions on 3–4 CKD patients and HD patients. To our knowledge, and as a result of our search, there are no clinical trials investigating chrononutrition interventions in patients with CKD to date.

Given the limited number of eligible studies, we included some intervention studies conducted in the HD population to contextualize the potential effects of chrononutrition strategies on nutritional status and disease progression in CKD patients. These studies examined the effects of supplement administration at different times of the day and/or on hemodialysis versus non-hemodialysis days. However, they were not designed with a chrononutrition approach, and as a result, their analyses and findings did not include specific time-related variables. Additionally, we incorporated examples from non-CKD populations to provide further insight into how chrononutrition principles might be applied to the management of CKD.

### 3.1. Eating Windows

Eating windows refer to the period of daytime eating duration (hours elapsed from the first to the last meal of the day). Short feeding windows (<10 h) have shown potential benefits in improving insulin sensitivity, reducing inflammation, and enhancing blood lipid profiles in certain individuals [54]. Medium feeding windows (11–12 h) tend to be neutral or slightly beneficial for metabolic health, depending on diet quality and meal timing. On the other hand, long feeding windows (>13 h), especially when they include late or nighttime meals, have been associated with negative effects such as circadian rhythm disruptions, increased risk of obesity, metabolic dysfunction, and impaired sleep quality in some individuals [55]. It is important to note that these effects can vary significantly between individuals due to differences in genetics, lifestyle, and underlying health conditions [54] such as CKD.

Given this evidence, shortening the eating window has emerged as a chrononutrition strategy in populations with nutrition-related excess diseases. Approaches such as time-restricted feeding (TRF) and intermittent fasting have shown promising benefits in individuals with conditions like obesity and diabetes, improving metabolic health markers and supporting weight management [56].

Due to ethical considerations, studies on fasting in CKD are predominantly observational on Middle Eastern populations, who practice Ramadan fasting (28–30 days of sunrise-sunset 12–19 h fasting windows).

On patients with early CKD, Karatas et al. found that eGFR was 14.8 mg/min/m^2^ higher after Ramadan in the group who fast compared with those who did not (*p* < 0.0001) [57]. A cross-sectional study on patients with type 2 diabetes and stable CKD stage 3 found no significant differences in clinical or biochemical parameters between fasting and non-fasting individuals, suggesting that such patients may be able to fast safely during Ramadan [58].

A prospective observational study followed up 28 adult early CKD patients before, during and after Ramadan fasting. The results showed that four patients worsened kidney function during fasting (two improved later and two continued to have elevated creatinine). The advanced CKD stage was the only factor associated with worse renal function [59,60]. Nevertheless, another study that included ninety-four 3–5 CKD patients, only advanced age was found to be associated with a ≥25% drop in eGFR after Ramadan in the fasting group, but was not associated with increased risk of declining renal function [61]. Regarding dialysis population, a 12-week, multicenter, prospective observational study evaluated the effects of intermittent fasting during Ramadan on 87 HD patients. Nutritional and functional status were assessed before Ramadan, during the fourth week, and four weeks after Ramadan. Results showed significant reductions in body mass index, interdialytic weight gain, waist circumference, fat tissue mass, and body fat percentage during Ramadan, with no significant changes in lean tissue mass. Serum phosphate levels improved, while albumin, urea, and creatinine levels decreased. In spite of day fasting, patients managed to maintain energy and protein intake, with improvements in handgrip strength during and after Ramadan. Overall, intermittent fasting during Ramadan was associated with temporary, non-detrimental changes in nutritional status in HD patients [62].

A pilot study in patients with overweight or obesity and CKD stages 3–4, assigned them to either a time-restricted feeding 8 h window (8 HW) group or a control diet (CD) which was a high-quality low-protein diet with no restrictions on what time they could eat each day, following their daily routines. During a follow-up of 12 weeks, found a significant improvement in eGFR in the 8 HW group (3.1 ± 5.3 mL/min/1.73 m^2^) compared to the CD group (−0.8 ± 4.4 mL/min/1.73 m^2^). In addition, the 8 HW group had a significant decrease in uric acid (70.8 ± 124.2 mmol/L), along with an increase in total protein (1.7 ± 2.5 g/L). The 8 HW group also showed a significant reduction in body weight (−2.8 ± 2.9 kg) compared to the control group, while body composition indicated a similar decrease in body fat mass, fat-free mass, and body water. Additionally, 8 HW shifted the gut microbiota in a positive direction [63].

Studies specifically designed with a chrononutrition focus could help uncover key aspects affecting nutritional status in CKD patients, offering insights into timing-related impacts on health outcomes. Stronger evidence in other populations could help elucidate those findings and possibly improve the CKD patient’s nutritional status.

### 3.2. Meal Timing Across the Day/Night Cycle

The responsiveness of tissue clocks to time cues varies. When these signals are received at irregular times, phase misalignment may occur. For instance, eating during the light phase helps maintain alignment, while consuming meals in the dark phase can disrupt systemic metabolic regulation [4]. A single meal consumed at different times of day can trigger different metabolic responses due to circadian fluctuations in energy absorption and utilization [64].

Some interventions have been designed for patients with metabolic alterations with positive results. Sutton et al. demonstrated that early eating (finishing eating before 3:00 PM) resulted in substantial improvements in insulin levels, insulin sensitivity, blood pressure, and oxidative stress levels in men with prediabetes [30].

In a clinical trial on people with obesity evaluating the role of food timing in weight-loss effectiveness among 420 individuals in a 20-week weight-loss program, late lunch eaters (those eating lunch after 3:00 p.m.) lost less weight and had a slower weight-loss rate compared to early eaters (*p* = 0.002). Despite similar energy intake, dietary composition, estimated energy expenditure, appetite hormones, and sleep duration between the groups, late eaters were more likely to be evening CT, consumed less energy at breakfast, and were more likely to skip breakfast (all *p* < 0.05). These findings suggest that meal timing, independent of caloric intake or expenditure, may influence weight-loss outcomes [65]. Growing evidence from observational studies shows that later meal timing is associated with metabolic disturbances [66].

A retrospective cohort in Japan, followed for 3.4 years, included 26,764 subjects over 40 years old (without baseline proteinuria or eGFR < 60 mL/min). The study evaluated exposure to unhealthy dietary habits and assessed the development of proteinuria as a prognostic factor for CKD. It was found that participants who ate late dinner (within 2 h before bedtime) had a 12% higher risk (hazard ratio [HR] 1.12, 95% confidence interval [CI] 1.02–1.22) and those who regularly skipped breakfast had a 15% higher risk (HR 1.15, 95% CI 1.01–1.31) of developing proteinuria [67].

The influence of day/night eating has also demonstrated associations with diet quality and nutritional status. Individuals with an evening CT tended to have less healthy eating habits, which can contribute to metabolic disorders [68,69,70]. Remarkably, there is no information to date about specific studies examining the role of chrononutrition in CKD populations, HD, PD, or transplant patients.

### 3.3. Midpoint of Eating

The eating midpoint is defined as the midpoint of the eating window. Adults with latest eating midpoints (in the second and third tertiles) showed a higher prevalence of elevated fasting glucose compared to those with an earlier eating midpoint (first tertile). Specifically, individuals in the second tertile had a 30% higher incidence rate ratio (IRR, 1.30; 95% confidence interval [CI], 1.07–1.59), and those in the third tertile had a 65% higher rate (IRR, 1.65; 95% CI, 1.22–2.22) for elevated fasting glucose [71].

A cross-sectional study based on NHANES 2013–2020 found a strong correlation between an earlier eating midpoint and a reduced incidence of CKD. Eating early in the day may potentially improve renal outcomes in patients with diabetes while a late midpoint is associated with increased risk of diabetic kidney disease [72].

Despite these findings, there is currently limited evidence on the impact of the eating midpoint in other CKD populations, such as subjects on renal replacement therapy.

### 3.4. Eating Jet Lag

Irregular or inconsistent meal schedules may misalign with the circadian rhythm, leading to metabolic disturbances. To study the meals regularity, the eating jet lag has been used. This parameter can be estimated by the difference between the midpoint of eating between weekends and weekdays. A positive association between eating jet lag and Body Mass Index (BMI) (*p* = 0.008) has been found; the threshold of eating jet lag of longer than 3.5 h could significantly increase BMI [53].

Eating jet lag is relevant in CKD as medical therapies modify patients’ day schedules. For example, in HD, changes in the individual’s routines between weekdays and weekends depends on the HD session day, compared to patients on PD whose treatment is the same every day of the week.

Burrowes et al. studied dietary patterns and eating habits in HD patients, including frequency of meals and snacks consumed daily during dialysis treatment. Less mealtimes were observed on HD than non-HD days (48% vs. 64% eat 3–5 times/day). Additionally, 11% of patients reported eating nothing or only one meal a day on HD days, compared to 5% on non-HD days [73].

HD therapy has been reported to influence meal timing. In a study of 142 subjects undergoing HD three times a week, it was observed that on dialysis days, only 6% of the subjects consumed all three main meals. Most patients ate either one (37%) or two (44%) meals per day, and 13% reported not consuming any of the three main meals on the HD day. In the same study, meal patterns (regular breakfast, lunch, and dinner) were compared between patients from the morning vs. afternoon shift of HD sessions. Patients in the afternoon shift had a higher breakfast frequency compared to individuals in the morning shift (63% vs. 16%; *p* < 0.001). On the other hand, patients undergoing morning dialysis had a lower lunch frequency (62% versus 13%; *p* < 0.0001) than those with afternoon dialysis. Having dinner was associated with the morning dialysis session, hypertension, and higher protein intake assessed by normalized protein equivalent of total nitrogen appearance (nPNA) [74]. This observation is similar to findings in other populations: night shift workers who had later meals showed higher total protein intake (7.8 vs. 4.7 g/kg/day *p* = 0.02) compared with day shift workers who eat earlier [75].

### 3.5. CT-Based Nutrition Intervention

It has been demonstrated that individuals with evening CT tend to consume the majority of their caloric intake in the second half of the day and exhibit less healthy eating patterns, such as lower consumption of fruits and vegetables and higher intake of ultra-processed foods [76]. In contrast, individuals with a morning CT showed positive associations between BMI and factors such as the timing of the first meal, morning latency, and the eating midpoint, while a negative association was observed with evening eating [28].

As a result of these associations, dietary interventions based on CT have begun to be studied. Dietary interventions tailored to CT have been shown to be effective for weight loss. A randomized clinical trial comparing a control diet with a CT-adjusted diet found that, although both groups improved anthropometric parameters, the CT-adjusted diet group achieved significantly greater reductions in total body weight percentage, BMI, and waist circumference (*p* < 0.010 for all). However, the effects on clinical parameters were less pronounced [77].

In addition to their impact on weight loss, the effect of such interventions is currently being studied in other parameters, such as cardiometabolic health and gut microbiota [78].

In patients with CKD, those associations have not been evaluated yet, which carries a challenge due to the various previously described chronodisruptors.

## 4. Discussion

### 4.1. Chrononutrition Interventions and Nutritional Challenges on CKD

As previously shown, chrononutrition aspects may play a crucial role in nutritional status outcomes. Nevertheless, there are few studies on chrononutrition in humans with CKD, and even fewer that explore the chrononutrition habits of people with different stages of the disease and renal replacement therapies. Each of these conditions present distinct lifestyle circumstances and influencing factors, highlighting the need for further exploration in the field of chrononutrition.

The CKD population exhibits diverse metabolic differences and nutritional goals across its various stages. This diversity makes the chrononutrition approach not only intriguing but also adaptable, providing the opportunity for tailored interventions that address the specific needs of CKD patients and potentially enhance their effectiveness.

Reaching an optimal nutritional status is a major challenge for the CKD population. According to this, a critical concern is the high prevalence of PEW, affecting 11–54% of non-dialysis CKD stages 3–5 and 28–54% of dialysis patients [79], significantly increasing the risk of mortality and adverse outcomes such as infections, hospitalizations, and disease progression [80,81].

PEW is a complex syndrome resulting from the interaction of inflammatory, uremic, and nutritional factors. Traditionally, its development has been associated with conditions such as anorexia, metabolic disorders, depression, nutrient losses, and dietary restrictions. However, emerging evidence suggests that novel factors could also play a significant role. These include sensory impairments, such as altered taste perception [82] and olfactory deficits [83], alterations in gut microbiota [84], and eating disorders such as pica [85].

Some of these novel alterations are influenced by circadian variations [86,87], as an example, taste sensitivity could exhibit daily, monthly, or yearly temporal patterns [87], however it has been poorly explored, particularly regarding their implications on nutritional status in CKD.

Therefore, it is essential to emphasize that nutritional intervention plays a critical role in the management of these patients. Preventing and managing PEW in all clinical stages is essential to decrease mortality and improve quality of life. Strategies include dietary optimization, alongside nutritional supplementation [88]. Precision nutrition approaches and targeted interventions tailored to individual needs show promise in mitigating PEW and slowing CKD progression [81].

Regarding energy intake, the primary challenge is to maintain a neutral to positive nitrogen balance and adequate body composition, therefore, the guidelines recommend diets providing 25–35 kcal/kg/day for patients in stages G1 to G5 of CKD [88].

In the early stages of CKD and also transplant patients, an important challenge is to prevent or treat excessive energy intake, which increases the risk of insulin resistance, diabetes, and cardiovascular disease [89]. In these patients, along with energy restriction interventions, chrononutritional strategies like shortening feeding windows might have beneficial effects for metabolic control, as has been shown in other conditions such as diabetes or obesity, effects that impact on slowing disease progression [63]. Some authors, based on the results of observational studies on Ramadan fasting, suggest that early-stage CKD patients can fast with careful monitoring [90] as well as transplant patients [91].

An earlier eating midpoint, which has been associated with promoting metabolic health, may benefit kidney transplant recipients by potentially improving insulin resistance and reducing the risk of post-transplant metabolic complications such as hyperlipidemia [92].

Conversely, a short feeding window in patients who live with advanced CKD or dialysis and at high risk of protein-energetic wasting [93], could be associated with reduced nutrient intake throughout the day, limiting the opportunity to eat. Therefore, it may not be the optimal strategy for patients who are at high risk of PEW or already experiencing it.

Studies with short eating windows must ensure total caloric intake; however, it is important to consider that advanced CKD patients often experience gastrointestinal symptoms (nausea, vomiting, diarrhea, stomach discomfort, constipation) (Biruete A, 2021) [93], which could be exacerbated by short windows, requiring meals with high energy density.

Due to ethical considerations, studies on fasting in CKD are predominantly observational, which presents a limitation. Nevertheless, observational studies specifically designed with a chrononutrition focus could help uncover key aspects affecting nutritional status in CKD patients, offering insights into timing-related impacts on health outcomes. Stronger evidence in other populations could help elucidate those findings and possibly improve the CKD patient’s nutritional status.

Another challenge for CKD patients is their protein intake. With the aim of reducing uremia, uremic toxins, hyperfiltration, and improving hemodynamics and renal function, the KDOQI guidelines recommend a limited protein intake for non-dialysis patients in stages G3 to G5 (0.55–0.6 g/kg/day for individuals without diabetes and 0.6–0.8 g/kg/day for those with diabetes) and adjusted intake of 1.0–1.2 g/kg/day for HD and 1.0–1.3 g/kg/day for PD [94].

Evidence in non-CKD populations has demonstrated that protein intake and its distribution across meal times play a key role in muscle protein synthesis. It is stimulated by approximately 3.0 g of leucine, corresponding to 30–35 g of high-quality protein, with the anabolic response lasting about 2.5 h. Interestingly the most effective intervention for muscle activation occurs when protein is administrated in the first meal after an overnight fast [95]. Therefore, consuming one protein-rich meal is essential when daily intake is limited. This is a chrononutrition strategy that could be particularly promising for patients with risk of protein-energy wasting, either or not undergoing dialysis therapies.

In addition, chrononutrition is a promising approach that could be tailored to each specific dialysis modality, as the schedules of different dialysis therapies significantly impact dietary intake [73,74]. Hypothetically, in HD patients, aligning nutrient intake with optimal metabolic windows such as an earlier eating midpoint might support metabolic stability, but the effect of the different HD shifts could be a challenging variable to explore. As previously described, patients’ eating patterns vary depending on their HD schedule, frequently skipping meals corresponding to the HD shift [73,74]. Interventions with nutrient prescriptions in specific mealtimes aiming to minimize the eating jet lag could potentially enhance metabolic stability and optimize the nutrients and caloric intake. Regarding oral supplement interventions, evidence has demonstrated that administering the same supplement at different times can produce different results, probably related to better alignment to dialysis schedules. In a randomized trial on malnourished HD patients, intradialytic oral nutrition supplementation improved nutritional status compared to interdialytic oral nutrition supplementation [96]. This mechanism could be applied to achieve nutritional goals in CKD patients while also impacting chronobiological variables such as sleep quality [97].

Patients undergoing PD deserve special consideration. PD therapy itself might alter appetite [98] because of the dialysis fluid in the peritoneal cavity, and the continuous administration of glucose through dialysate that provides a strong satiety stimulus. However, its impact on chronobiology and chrononutrition is a topic that warrants further investigation. Also, meal patterns could be altered according to the PD schedule. Patients undergoing continuous ambulatory PD perform exchanges every 4 h [99] and could benefit from tailored interventions that align with their frequent exchange schedule to optimize nutrient intake and metabolic outcomes. Patients on automated PD typically adhere to nocturnal dialysis schedules [99], making interventions aimed at minimizing circadian misalignment beneficial for improving glycemic control and reducing cardiovascular risk. Short or medium eating windows could potentially enhance these effects, with further research needed to determine whether a longer morning or evening window yields better metabolic outcomes. Such approaches would allow for a circadian catabolic phase that complements their overnight treatment regimen.

Notably, no chrononutrition interventions have been reported for patients undergoing PD or those with kidney transplants. Future studies should focus on evaluating the specific effects of administering dietary supplements at different times of the day, taking into account individual CT and other chronobiological factors. Such research could provide evidence-based guidelines for tailoring chrononutrition interventions, ultimately improving health outcomes in patients with CKD and other chronic conditions.

Certain chrononutrition strategies may also interact with common pharmacological treatments for CKD, (such as prolonged fasting or extreme caloric restriction at specific times), potentially affecting drug absorption or metabolism. For transplant patients, the morning fasting is important due to the pharmacokinetics of the medical therapy. As an example, findings from a study revealed significant tacrolimus circadian variations under fasting conditions. When the dose was administered in the morning during a fasted state, there was a 45% higher peak concentration and a 20% greater area under the curve compared to doses given at other times [100]. These results suggest that the timing of administration and fasting status can substantially influence absorption rates and overall drug exposure, which is important to consider in the management of patients with CKD.

A final strategy to explore is designing chrononutrition interventions considering the CT. This could potentially enhance adherence and improve metabolic outcomes, offering a personalized approach to dietary management that aligns with patients’ natural rhythms, highlighting the potential for diet to either mitigate or worsen chronodisruption.

### 4.2. Considering Potential Risk on Chrononutrition Interventions

While the benefits of chrononutrition in managing CKD are becoming increasingly evident, it is equally important to consider potential risks or adverse effects associated with this approach. Misalignment between prescribed meal timing and individual circadian rhythms, particularly in patients with irregular sleep patterns, could lead to metabolic disturbances or reduced adherence to dietary recommendations [4,101]. Additionally, restrictive timing windows for food intake might unintentionally result in inadequate caloric or nutrient intake, exacerbating malnutrition risk—a prevalent concern in CKD patients.

Future studies should carefully evaluate these risks, ensuring that chrononutrition protocols are tailored to individual patient needs and clinical conditions to maximize safety and effectiveness.

### 4.3. Practical Recommendations

In nutritional counseling, an assessment of chrononutrition aspects could be incorporated to gain a comprehensive understanding of the patient’s eating habits. Beyond assessing meal timing through 24 h recalls or food frequency questionnaires, it is recommended to integrate tools that analyze chronobiology-related factors, such as patient’s CT to identify individual patterns of activity and rest, examining the regularity of meal timing, and exploring differences in the number and size of meals consumed on workdays versus non-workdays. These elements are crucial for designing personalized strategies that optimize the alignment between biological rhythms and nutritional interventions, aiming to improve both nutritional status and disease management.

Nutritional interventions, in addition to addressing clinical aspects and dietary preferences, should also consider chronobiological factors, including biological rhythms and chronodisruptors. By aligning meal schedules with these rhythms, interventions can be optimized to enhance their effectiveness and support overall health [3]. Incorporating the patient’s CT and potential eating jetlag into the design of dietary plans may enhance adherence and efficacy. For instance, aligning meal and supplement timing with the patient’s biological rhythms could optimize nutrient absorption and metabolic responses.

Also, we need to develop monitoring approaches or adapt the available ones, such as the use of food diaries, smartphone applications, wearable devices, and validated tools for assessing adherence to chrononutrition interventions (including biomarkers) in clinical practice.

### 4.4. Interdisciplinary Perspective of Chrononutrition in CKD

**Figure 3:**
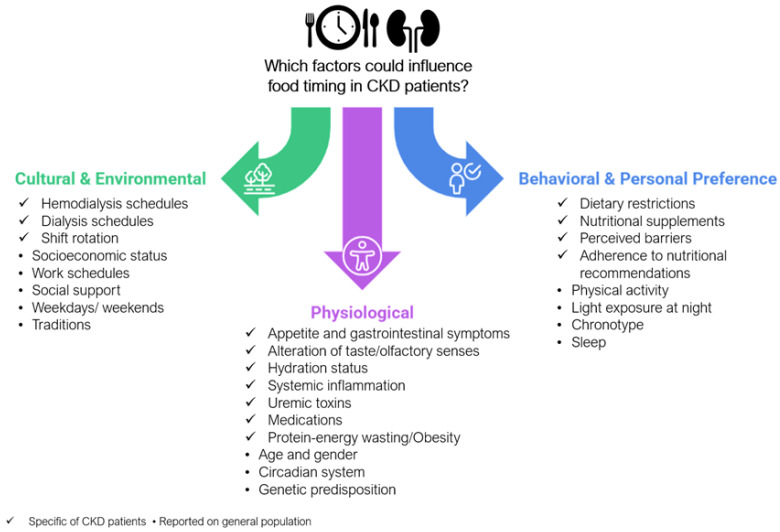
Possible food timing determinants in CKD. The figure illustrates the primary categories and determinants of food timing including cultural and environmental, behavioral and personal preferences, and physiological factors. Within each category, relevant factors are listed; Corresponding potential determinants in CKD patients and also that have been studied in the general population,. This categorization highlights how food timing influences can be tailored to the specific needs and challenges faced by CKD patients.

There are three major determinants of meal times for the general population: environmental and cultural, behavioral and personal preferences, and physiological determinants [50]. In this context, some characteristics well-known in people who live with CKD can be added to these categories as potential determinants of food timing (Figure 3). Nevertheless, these factors and their relevance have been insufficiently explored in the context of people living with CKD, highlighting the need for further investigation into how they might influence meal timing in this population.

Emerging evidence suggests that integrating complementary interventions, such as scheduled physical activity and regulation of sleep patterns, could further optimize the observed benefits. Regular physical exercise, particularly when aligned with circadian rhythms, has been shown to improve metabolic function, reduce inflammation, and enhance nutrient utilization efficiency [101]. Similarly, sleep quality and duration play a fundamental role in energy homeostasis and the modulation of appetite-regulating hormones.

Future research combining chrononutrition strategies with exercise and sleep interventions could provide a more effective, holistic approach to nutritional management in CKD patients. Additionally, study designs that consider the interaction between these factors would allow for a better understanding of their synergistic effects on clinical outcomes.

### 4.5. Limitations

The scarcity of studies on chrononutrition in CKD represents one of the primary limitations of this review. This gap is largely due to the novelty of the topic and the fact that many available chrononutrition studies exclude individuals with CKD as part of their inclusion criteria. Consequently, evidence specific to this population remains limited, restricting the depth of analysis and the robustness of conclusions. Given this scenario, a narrative review was chosen as the most appropriate format to explore the available knowledge, despite the inherent limitations that carry this approach. Additionally, the heterogeneity of the available evidence—in terms of methodologies, populations, and outcomes—makes it difficult to draw broadly generalizable conclusions.

Nevertheless, this represents a great opportunity for the scientific community to develop robust observational and interventional studies focused on CKD populations, incorporating principles of chronobiology and chrononutrition. In doing so, we can aim to generate high-quality evidence capable of informing clinical practice guidelines, ultimately improving the care and quality of life of patients with CKD.

## 5. Conclusions

Understanding chrononutrition behaviors in CKD patients is crucial because it remains largely unexplored. The impact of chrononutrition habits on nutritional status is unclear. Additionally, there is a lack of data on the CT of people who live with CKD and its association with diet quality and nutritional status. Addressing these gaps is essential for developing targeted interventions that can improve the health outcomes of CKD patients. Additionally, it will be interesting to include elements of chronobiology in interventions such as diet, supplementation, and exercise in this kind of patient. This approach could explore whether integrating chronobiological principles might enhance the beneficial effects of these interventions, aligning them more closely with their respective objectives. By tailoring interventions to the circadian rhythms of CKD patients, we could potentially improve their efficacy and overall health outcomes.

## Supplementary Materials

The following supporting information can be downloaded at: https://www.mdpi.com/article/10.3390/nu17030389/s1, Table S1: Research strategy.

## Abbreviations

The following abbreviations are used in this manuscript:

CKD	Chronic kidney disease
SCN	Suprachiasmatic nucleus
CT	Chronotype
GFR	Glomerular filtration rate
HD	Hemodialysis
PD	Peritoneal dialysis
TRF	Time-restricted feeding
8 HW	8 h window
CD	Control diet
PEW	Protein-energetic wasting
BMI	Body Mass Index
nPNA	Normalized protein equivalent of total nitrogen appearance
