# Supplementary material for: Chrononutrition in Chronic Kidney Disease
Source: Nutrients. 2025 Jan 22;17(3):389. doi: 10.3390/nu17030389 (PMC11820925; doi:10.3390/nu17030389)
Supplement: Supplementary file 1 [file nutrients-17-00389-s001.zip › nutrients-3424164-supplementary.pdf]

Supplemetary TableS1. Research strategy.

	P (Population)	I Intervention)/Exposure	O (Outcome)
Definition	Adult patients with CKD at different stages (early stage, end-stage, HD, DP or transplant)	Chrononutrition (eating/fasting windows, timing of food/supplement intake, chronotype-based meal timing).	Nutritional status
Keywords	"renal insufficiency, chronic"[MeSH Terms]<br>"kidney failure, chronic"[MeSH Terms]<br>"CKD"[All Fields]<br>"chronic kidney disease"[All Fields]<br>"dialisys"[All Fields]<br>"haemodialysis"[All Fields] OR "renal dialysis"[MeSH Terms] OR ("renal"[All Fields] AND "dialysis"[All Fields]) OR "renal dialysis"[All Fields] OR "hemodialysis"[All Fields]<br>"peritoneal dialysis"[MeSH Terms] OR ("peritoneal"[All Fields] OR "dialysis"[All Fields]) OR "peritoneal dialysis"[All Fields]<br>"peritoneal dialysis, continuous ambulatory"[MeSH Terms]<br>"Peritoneal Dialysis"[MeSH Terms]<br>"Renal Dialysis"[MeSH Terms]<br>OR "kidney transplantation"[MeSH Terms] OR ("kidney"[All Fields] OR "transplantation"[All Fields]) OR "kidney	"chrononutrition"[All Fields] OR "Meals"[MeSH Terms] OR ("Meals"[MeSH Terms] OR "Meals"[All Fields] OR ("meal"[All Fields] AND "time"[All Fields]) OR "meal time"[All Fields]) OR (("Meals"[MeSH Terms] OR "Meals"[All Fields] OR "meal"[All Fields]) OR "timing"[All Fields] OR "timings"[All Fields])) OR (("food"[MeSH Terms] OR "food"[All Fields]) AND ("timely"[All Fields] OR "timing"[All Fields] OR "timings"[All Fields])) OR "Dietary Supplements"[MeSH Terms] OR ("Dietary Supplements"[MeSH Terms] OR ("dietary"[All Fields] OR "Intermittent Fasting"[MeSH Terms] OR "Fasting"[MeSH Terms] OR "eating window"[All Fields] OR ("Intermittent Fasting"[MeSH Terms] OR	("Nutritional Status"[Mesh] OR "Protein-Energy Malnutrition"[Mesh] OR "Body Composition"[Mesh] OR protein-energetic wasting OR PEW OR protein energetic waste)

	transplantation"[All Fields] OR ("renal"[All Fields] OR "transplant"[All Fields]) OR "renal transplant"[All Fields]	("intermittent"[All Fields] AND "Fasting"[All Fields]) OR "Intermittent Fasting"[All Fields] OR ("time"[All Fields] AND "restricted"[All Fields] AND "feeding"[All Fields]) OR "time restricted feeding"[All Fields]) OR ("mid-point"[All Fields] AND ("eating"[MeSH Terms] OR "eating"[All Fields])) OR ("Chronotype"[MeSH Terms] OR "Chronotype"[All Fields] OR "chronotypes"[All Fields] OR "chronotyped"[All Fields]) OR "Chronotype"[MeSH Terms] OR (("eating"[MeSH Terms] OR "eating"[All Fields]) AND ("jet lag"[MeSH Terms] OR ("jet"[All Fields] AND "lag"[All Fields] AND "syndrome"[All Fields]) OR "jet lag syndrome"[All Fields] OR ("jet"[All Fields] AND "lag"[All Fields]) OR "jet lag"[All Fields]))	

Filters: (clinicaltrial[Filter] OR observationalstudy[Filter] OR randomizedcontrolledtrial[Filter] OR pilotstudy[Filter]) AND (humans[Filter]) AND (english[Filter] OR spanish[Filter])
